# Community violence and academic achievement: High-crime neighborhoods, hotspot streets, and the geographic scale of “community”

**DOI:** 10.1371/journal.pone.0258577

**Published:** 2021-11-10

**Authors:** Daniel T. O’Brien, Nancy E. Hill, Mariah Contreras

**Affiliations:** 1 School of Public Policy and Urban Affairs, Northeastern University, Boston, MA, United States of America; 2 Boston Area Research Initiative, Northeastern & Harvard Universities, Boston, MA, United States of America; 3 Harvard Graduate School of Education, Harvard University, Cambridge, MA, United States of America; 4 Eliot-Pearson Department of Child Study, Tufts University, Medford, MA, United States of America; 5 Department of Urban and Environmental Policy and Planning, Tufts University, Meford, MA, United States of America; Montclair State University, UNITED STATES

## Abstract

Numerous studies have demonstrated a negative relationship between community violence and youth academic achievement, but they have varied in their geographic definition of “community,” especially as it relates to proximity to students’ residences. We extend this by considering the independent relationships between academic achievement and violent events (from 911 dispatches; e.g., gun shots) at the neighborhood (i.e., census tract) and street-block levels. We use data from standardized Math and English Language Arts (ELA) tests from Boston, MA for 2011–2013. Exposure to community violence was partially independent between streets and tracts, with some students living on low-crime streets in high-crime neighborhoods or high-crime streets in low-crime neighborhoods. Initial regression models found that differences in a neighborhood’s violent crime predicted up to a 3% difference in test scores on both Math and ELA tests. Students living on high-crime streets scored an additional 1% lower than neighbors on safer streets. Subsequent models with student-level fixed effects, however, eliminated these relationships, except for the effect of neighborhood-level violence on Math scores. These findings suggest that future work should consider community violence at both geographic scales, but that in this case the impacts were only consistent at the neighborhood level and associations at the street level were seemingly due to spatial segregation of households.

## Introduction

Exposure to community violence can have substantial deleterious impacts on student academic achievement [e.g., 1–4], highlighting how much where one lives can matter. Researchers have conducted many studies on the subject, but they have interpreted the geographic scale of “community” in two different ways. The majority of studies have focused on how violence in one’s “neighborhood,” approximated using census geographies, impacts academic achievement [e.g., 5]. Others have looked at exposure to violence at more localized scales, like the street block [e.g., 4]. These two lines of work have largely been independent, leaving ambiguity as to how young people are exposed to community violence at each of these geographic scales and their relative importance for academic outcomes.

There are reasonable arguments for why either streets or neighborhoods might be critical in the community violence-academic achievement relationship. It is well known that crime varies markedly from block to block within a neighborhood [6–8] and that each individual resident tends to spend the greatest amount of time on the handful of streets near his or her home [as confirmed by electronic records that track mobility and activity; 9–11]. This would suggest that he or she would have more exposure to events in that narrowly defined region. On the other hand, the totality of crime in a neighborhood may better reflect the broader social context experienced by a young person. To date, no study has tested the impacts of community violence on academic achievement at these two geographic scales simultaneously, meaning their relative importance remains unknown.

The current study presents an initial effort to simultaneously consider how violent crime at the street and neighborhood levels relates to the academic achievement of the youth living there. We do so by analyzing the geographic distribution of standardized test scores for Boston Public Schools (BPS) students across three school years, pursuing three main questions. First, we examine the proportion of students who live in high-crime neighborhoods or on high-crime streets (or “hotspots”). Importantly, we identify the prevalence of cases in which one geographic scale is high-crime and the other is not (e.g., a student living on a hotspot in an otherwise low-crime neighborhood), thereby capturing how each scale might provide a different view on a given individual’s exposure to community violence. Second, we use regression models to quantify the independent associations between street- and neighborhood-level community violence and academic achievement. Third, we leverage the longitudinal nature of the data to run models that include student-level fixed effects, thereby robustly testing the impact of community violence at each of the geographic scales on academic achievement. Before proceeding to the data and analysis, the following subsections: situate this study within the existing literature on community violence and academic achievement; compare work at the neighborhood and street block levels; and describe in greater detail the interpretation of our two-stage analysis.

### Community violence and academic achievement

A sizable body of work has found that students living in communities with higher levels of violence have lower academic achievement than their peers, as measured through coursework and standardized tests [1–3, 12–18]. There is also evidence that it can lower school engagement overall by increasing absenteeism [19]. There are three overarching theories as to why this association exists: (1) trauma from exposure to violence interferes with learning; (2) socialization to norms associated with a violent context detract from school-oriented behavior; and (3) spatial segregation, or the potential that certain families who live in high-crime communities also experience other challenges that lead to lower academic achievement in youth. We summarize these in turn.

Youth who have been exposed to violence in their community—even if they heard about it rather than witnessing it—tend to experience trauma [20, 21]. Arguably, this is not only a reaction to the violence itself but to the possibility that one lives in a threatening context. This trauma can then lead to dysregulated and externalizing behaviors, such as delinquency and aggression [22], which distract or interfere with academic performance [2, 3, 16]. It can also give rise to internalizing behaviors [23–25], such as depression and a subsequent retreat from school and academic work [26, 27]. In addition to this work on the long-term impact of trauma, an allied set of studies has uncovered acute effects on cognitive assessments and academic performance shortly after crime events near a student’s home [4, 12].

A second set of explanations for the community violence-academic achievement relationship implicate socialization processes in high-crime communities. Anderson [28] describes how youth in high-crime communities adopt norms and behavioral tendencies that make it easier to survive and succeed in a violent context. These behaviors, however, are not necessarily aligned with success in the classroom. Likewise, Harding [29, 30] discusses how students in such contexts experience competing cultural frames. On the one hand, they are encouraged to perform well in school, but on the other they receive contradictory messages and norms from older peers that deemphasize the value of schooling in favor of “street smarts” for surviving in high-risk, high-crime neighborhoods. There is also evidence that aggressive law enforcement tactics sometimes implemented in high-crime neighborhoods, including racial profiling, may result in youths’ developing tendencies to contest and resist institutional authorities, including school personnel [31]. These processes are not necessarily direct responses to exposure to violent crime, but instead reflect social dynamics that can arise in a community with high levels of violence that in turn lead to habits that detract from academic achievement.

A third possibility is that the association between academic achievement and community violence is an artifact of other causal factors. The simplest such possibility is that violent crime is correlated with other social and environmental factors that have more proximal impacts on academic achievement. This is a form of omitted variable bias and can be addressed through the incorporation of appropriate correlates into a model. More conceptually and analytically challenging is *spatial segregation*, wherein certain crucial characteristics and experiences of a household are correlated with the conditions of the neighborhood in which they are likely to live. In the current case, this would seem possible through multiple pathways. For example, the Moving to Opportunity study found that many poor families who were enabled by federal funding to move to more affluent neighborhoods eventually returned to communities similar to those in which they lived originally [32, 33]. Some of this effect appears to be attributable to discrimination in higher-opportunity neighborhoods [34]. It might also be that they relocated to high-poverty (and, by association, higher-crime) neighborhoods because of financial, social, or logistical limitations that are also correlated with their ability to promote learning in their children. Similarly, a study in New Orleans, LA found that individuals with more health challenges were more likely to have relocated to high-poverty neighborhoods after Hurricane Katrina, independent of the poverty level of their home neighborhood before the storm, presumably because of their inability to seek out or afford more affluent or lower risk opportunities [35]. Conversely, families with the access, knowledge, resources, or foresight to procure and maintain housing in low-crime areas might apply the same assets to their children’s academic achievement. This would be particularly meaningful for low-income families whose housing options are often limited to high-crime areas.

Spatial segregation, whether voluntary or driven by structural limitations placed on households, can be a confounder when testing other hypotheses, creating artifactual correlations between household and neighborhood characteristics. For instance, households that are prone to lower academic achievement may by happenstance locate to communities with higher crime, but this does not guarantee that the latter is driving the former. This methodological challenge is referred to as *selection bias*. Some strong research designs have partially addressed the concern of selection bias. For example, multilevel models disentangle the effects of neighborhood- and individual-level factors [e.g., 36]. That said, although they control for observed features of students and families, they cannot do so for unobserved features that cluster across neighborhoods. The natural experiment approach adopted by Sharkey and colleagues [4, 12, 37] is effective for testing the acute effects of a violent event, but less so for evaluating the cumulative effects of a residential context with frequent violence. A strong methodology for verifying a causal effect of community violence on academic achievement is fixed effects models, but these require multiple years of data on the same students, severely limiting the number of studies that have been able to incorporate them [see 5 for an exception]. Executing any of these research designs, however, requires the researcher to determine how community is defined and the geographic scale at which violence is hypothesized to impact youth.

### The geographic scales of academic achievement and crime

Studies on academic achievement and community violence have varied considerably in how they have defined “community.” The three most common approaches have been to use: (1) neighborhoods, as approximated by census geographies [5, 12]; (2) the more narrow geographic scales of either the census block or street (i.e., face) block [4, 37, 38]; and (3) self-reports of exposure to violence within their communities [1–3, 15–17], leaving the definition of the community up to the interpretation of the respondent. Because of the geographic ambiguity of this last strategy, we set these studies aside, instead focusing on evidence from the other two. In doing so, it is interesting to note that studies using both neighborhoods and blocks or streets have found negative relationships between community violence and academic achievement. None, however, has analyzed the two scales in conjunction, leaving open questions regarding their independent association with and impact on academic achievement.

The multiple geographic scales used in the study of community violence and academic achievement closely parallels trends in urban science more broadly. Neighborhoods are historically the most common way to think about the impacts of one’s residential context, and census tracts are the most common approximation for neighborhoods, in part because they are readily available and have many other data sources associated with them [some studies have also used the smaller census block group; 5]. Conceptually, census tracts tend to have a size similar to the range that the average person indicates as “their neighborhood” in surveys and cognitive mapping exercises [39–41]. This makes them rough facsimiles of the residential context people actually experience, even if the boundaries do not always match the region with which they identify [39, 42].

The emphasis on the neighborhood-level scale entails two important assumptions, however, that have come into question. First, it is assumed that a census tract-sized region reflects a shared geographical space whose events and conditions impact all residents collectively and similarly. Each individual, however, tends to frequent a small number of the streets in her neighborhood, primarily those surrounding the home and connecting to major destinations [see 43]. If this first assumption does not hold, it then begs the question of whether neighborhoods are homogeneous environments, or whether street-to-street variations create markedly different experiences for residents of the same neighborhood. This assumption of within-neighborhood homogeneity has been especially undermined by work on *criminology of place*, which has argued that the street segment—not the neighborhood—is the ideal unit of analysis for observing the distribution of crime in a city. Indeed, across cities of various sizes and forms, only 4–6% of streets are responsible for 50% of crime events [6–8], prompting the maxim that most streets in high-crime neighborhoods are in fact safe, and even a low-crime neighborhood can harbor a “hotspot,” defined as a single street that has a chronically elevated crime rate.

The insights from criminology of place are especially pertinent for work on the community violence-academic achievement relationship. If the level of community violence on a street segment is partially independent of the aggregate level of violence across the neighborhood, then there are two different ways to specify a student’s level of exposure. Arguments could be made for why either or both geographic scales are associated with and consequential for academic achievement. For instance, direct or perceived exposure to violence is more likely for events nearby the home, and thus on the street block of residence. In contrast, socialization related to the prevalence of community violence might be anticipated to operate at the broader scale of the neighborhood. Even in absence of testing these specific mechanistic relationships directly, evaluating the geographic distinction is important when one considers the corollary implication that some youth live on hotspots in low-crime neighborhoods, and others on low-crime streets in high-crime neighborhoods; perspectives that address only one geographic scale will miss this nuance.

### Current study

Studies to date have presented evidence for the negative effect of community violence on student academic achievement at either the scale of the neighborhood (typically operationalized as census tracts) or the street block. None has disentangled effects at the two levels and compared them to each other, leaving a gap in our overall understanding of the community violence-academic achievement relationship. The current study fills this gap by analyzing the geographic distribution of standardized test scores for all students in Boston Public Schools (BPS) for three school years (2011–2012 through 2013–2014). We used 911 dispatches to identify violent events throughout the time period. The events were mapped to discrete addresses, from which we measured the level of violence at both the street and neighborhood levels. We used traditional calculations of crime rates for neighborhoods, as this is reflective of the overall penetration of violence in the community. We use counts for street segments, assuming that at such a tight geographic scale each individual event is meaningful (see Measures in Methods for more detail on these decisions). Further, drawing from the work on criminology of place we consider thresholds for classifying a street as a “low” or “high” hotspot.

The analysis is presented in three parts. Because this is the first study on the community violence-academic achievement relationship to consider both the neighborhood and street scales, we began by examining the extent to which students were exposed to crime at each of them. We focused specifically on the kinds of cases that have been highlighted by criminology of place—low-crime streets in high-crime neighborhoods, and hotspot streets in low-crime neighborhoods—as these reveal unique situations invisible to previous research. Second, we pursued a series of regressions that examined the associations between community violence at each of these geographic scales and academic achievement, controlling for contemporaneous correlates. Acknowledging that the there are many contexts and factors that impact learning and academic achievement beyond the curriculum, these correlates include individual and household characteristics as tracked by the school district as well as school-level fixed effects that account for differences in school climate (e.g., violence in the school; though noting that to the extent that aspects of school climate do vary across years this estimation strategy will capture these only as an average across the time period studied.). Third, we evaluated whether these associations were robust to selection bias, or the possibility that patterns of spatial segregation might create a correlation between a student’s lower performance on standardized tests and the tendency of a family to live on a street or in a neighborhood with higher levels of violent crime. We did so with a set of models that incorporate student-level fixed effects, leveraging the multiyear nature of our data and the fact that many students took tests in more than one year. Together, these analyses comprehensively illustrated the relationship between community violence and academic achievement at these two geographic scales.

## Methods

### Data sources

The research was approved by Northeastern University’s Institutional Review Board and permitted by the Boston Public Schools as it analyzed student administrative records. BPS provided student scores on standardized tests from the Massachusetts Comprehensive Assessment System (MCAS) for 2011–2012, 2012–2013, and 2013–2014. Students take MCAS tests in grades 3–8 and 10, receiving separate scores for Math and ELA, each on an 80-point scale (range: 200–280). Although these tests differ according to grade level, they are explicitly intended to track growth in these basic skills and are used in this way by the state and districts (see https://www.doe.mass.edu/mcas/growth/). The records also included student sex, race, poverty status (i.e., living under 185% of the poverty level, thereby qualifying for either free or reduced-price meals), English Language Learner status (current, former, or never), and enrollment in any Special Education programs (e.g., cognitive limitations, behavior problems, physical disability; dichotomous variable with 1 = enrolled in special education). This database contained 40,917 unique students that fit the following criteria: complete demographic information; a home address that could be geocoded; in a grade for which taking an MCAS test was appropriate, as opposed to taking it behind a grade level. The regression models were further limited to 23,569 students who took at least one subject test in at least two years and thus could be included in a fixed effects analysis. This subsample had nearly equivalent characteristics to the full sample (reported in Table 1).

**Table 1:** Characteristics of students, streets, and tracts for the total sample and the subsample of students included in the fixed effects models.

	Mean (SD) or Count (%)			Mean (SD) or Count (%)	
	Total Sample	Subsample		Total Sample	Subsample
**Students (*n* = 40,917 / 23,569)** ^a^					
*Sex*			*Race*		
Female	19,939 (49%)	11,471 (49%)	Asian	3,651 (9%)	2,154 (9%)
Male	20,978 (51%)	12,098 (51%)	Black	15,198 (37%)	8,368 (36%)
			15,544 (38%)	9,240 (39%)
*Grade* ^b^			Mixed / Other	885 (2%)	499 (2%)
3^rd^ Grade	7,257 (18%)	2,902 (12%)	Native Amer.	124 (<1%)	83 (<1%)
4^th^ Grade	5,262 (13%)	4,210 (18%)	White	5,515 (13%)	3,225 (14%)
5^th^ Grade	3,879 (9%)	3,189 (14%)			
6^th^ Grade	3,630 (9%)	3,101 (13%)	*Special Education* ^b^	7,796 (19%)	4,755 (20%)
7^th^ Grade	5,001 (12%)	4,119 (17%)			
8^th^ Grade	6,075 (15%)	4,593 (19%)	*ELL Status* ^b^		
10^th^ Grade	9,294 (23%)	1,454 (6%)	Current	10,328 (25%)	5,828 (25%)
			Former	6,991 (17%)	4,575 (19%)
*Poverty* ^c^			Never	23,598 (58%)	13,166 (56%)
Yes	31,426 (77%)	18,577 (79%)			
No	9,491 (23%)	4,992 (21%)			
*ELA MCAS Scores* ^d^	238 (15.56)	237 (15.37)	*Math MCAS Scores* ^d^	238 (19.19)	236 (18.42)
**Street Segments (*n* = 6,466 / 5,319)** ^a^					
Main Street	1,298 (20%)	1,083 (20%)			
*Predominant Zoning*					
Three-Family Res.	2,070 (32%)	1,832 (34%)	Exempt	182 (3%)	151 (3%)
Mixed Single- and Two-Family Res.	1,490 (23%)	1,212 (23%)	Condominiums	534 (8%)	419 (8%)
Commercial	153 (2%)	110 (2%)	Mixed-Use Commercial	234 (4%)	205 (4%)
Single-Family Res.	1,803 (28%)	1,390 (26%)			
*Violent Events (Annual)*	2.97 (6.24)	3.34 (6.63)			
**Census Tracts (*n* = 165 / 164)** ^a^ ^,^ ^e^					
*Violent Crime* ^f^	0.02 (0.99)	0.01 (1.00)	*Socioeconomic Status*	-0.01 (0.99)	-0.00 (0.99)
*Adult Acad*. *Attainment*	-0.02 (1.00)	-0.03 (1.00)	*Physical Disorder*	0.18 (0.58)	0.18 (0.58)
*Custodianship*	0.12 (0.86)	0.12 (0.85)			

^a^–Unique students, streets, and tracts represented in the full sample and in the subsample included in the regression and fixed effects models. Streets and tracts are a subset of the city’s 13,048 streets and 175 tracts with population.

^b^–In the earliest school year in which a student appears in the data.

^c^–Based on traditional criteria for qualification for free or reduced-price lunch.

^d^–On an 80-point scale from 200–280.

^e^–All tract descriptors are standardized constructs comprising one or more weighted items. All are centered at 0 with a standard deviation of 1 for the full population of tracts prior to analysis. Measures for 2013 are reported.

^f^–One standard deviation on average reflected an increase of 3.2 events/1,000 residents for prevalence of guns, 5.0 events/1,000 residents for private conflict, and 16.5 events/1,000 residents for public violence. The range was -1.49–2.98.

We measured violent events using 911 dispatches from 2011–2013. Over this time, the City of Boston had 1,681,616 dispatches that referenced an event at an address or intersection that could be uniquely identified in the list of known locations maintained by the City of Boston (locations were geocoded by the system at the time of dispatch using the City’s Street and Address Management service; 93% geocoding rate, with the remaining 7% lacking sufficient address or latitude-longitude information to be geocoded). The system utilizes a standardized list of case types to categorize all requests at the time of receipt. Previous work with these data used factor analysis to develop groupings of case types that act as indices of disorder and crime [44, 45]. We combined three indices of violence: *private conflict* arising from personal relationships (e.g., domestic violence); *public violence* that did not involve a gun (e.g., fight); and *prevalence of guns*, as indicated by shootings or other incidents involving guns. More detail on the case types included in each of these three measures and their frequencies are reported in the S1 File. Over the three years, there were 99,213 dispatches falling into one of these three categories, 96% of which were geocoded. This subset was used to calculate levels of violence for street segments, and all events were used to calculate tract violence levels.

We coordinated these two data sets using the Boston Area Research Initiative’s (BARI) Geographical Infrastructure for Boston [GI; 46], which links all land parcels (i.e., addresses) identified in the City of Boston’s Tax Assessments to U.S. Census TIGER line street segments (i.e., the undivided length of street between two intersections or an intersection and a dead end) and nests them within census tracts. 911 dispatches come with unique identifiers for parcels that link to the GI, but BPS records do not, for which reason we used a custom geocoder that appends these identifiers based on address information. The geocoding rate for the BPS records was 95% (with an 80% minimum match score). The final sample was distributed on 6,466 of the city’s 13,048 street segments and 165 of the city’s 178 census tracts (5,319 street segments and 164 census tracts for subset included in regression models).

### Measures

There are important considerations for how to best measure incidences of violent crime at the contrasting geographical scales of streets and tracts. For street segments, the logic is that the event occurred in the immediate vicinity of the student’s home and is thus perceived as immediately threatening. This would point to a count variable, considering every event on the street as a contribution to the experience of violent crime. This does not then require normalization by population (To ensure that this logic holds even for exceptionally long street segments wherein a student living on one end of the street might not perceive a violent event on the other end of the street, we re-ran the models excluding individuals whose streets were above 250 m (14% of students) and found all results to be the same; see S1 File). In contrast, studies on neighborhoods (or census tracts), traditionally normalize by population to create crime rates. This might seem inconsistent with measuring street-level violence in raw counts, but it is important to keep in mind that a student is not directly exposed to all events in their community. Instead, their knowledge of it is socially transmitted, and thus the perception of threat or the manner in which it the level of violence is embedded in local norms would likely be best proxied by the rate of victimization—e.g., “How many people do I know that have been victimized or witnessed crime?” As such, we maintain the traditional measurement strategy of population rate (drawn from American Community Survey 2009–2013 estimates).

The GI was used to create aggregate measures of violent crime. For streets this was done by tabulating the number of dispatches for events occurring at land parcels on the street that reflected private conflict, public violence, or prevalence of guns. These counts had a Poisson distribution (see Table 1), for which reason we log-transform them before analysis (log(count+1) to account for zeroes.). For tracts, we summed the dispatches for each of the three subcategories and then divided each by total population to calculate a rate. They were then normalized to create *z*-scores so they could be placed on a single scale and combined them in a weighted sum, according to the results of a factor analysis (weights = .88 - .89; see S1 File for full details). As a point of reference, an increase by 1 on this measure on average reflected an increase of 3.6 events/1,000 residents for prevalence of guns, 5.7 events/1,000 residents for private conflict, and 18.7 events/1,000 residents for public violence; the range was -1.49–2.98.

We drew on BARI’s Boston Data Portal (https://cssh.northeastern.edu/bari/boston-data-portal/) for a series of tract-level covariates known to be related to academic achievement. In nearly all cases these were multi-measure constructs intended to capture an overarching aspect of a neighborhood. BARI and BPS constructed them as an effort to specify the sources of geographic inequities in academic achievement. These included socioeconomic status (e.g., median household income), academic attainment of adults (e.g., proportion of adults with Bachelor’s degree), physical disorder [i.e., “broken windows," as drawn from 311 reports; 45], and custodianship [i.e., the tendency of residents to attend to issues in public spaces; 10]. The S1 File list the measures included in each of these constructs and their weighting (Work from an earlier project using these data on the neighborhood factors predicting academic achievement also examined the role of public health (e.g., premature mortality), proportion immigrant, and residential stability (e.g., median years living in the neighborhood), but found them to have no predictive power independent of the other factors [47]).

The GI also provided information about streets that can be relevant covariates for crime or other environmental features (e.g., noise) that could impact student thriving [48]. Leveraging these the models controlled for the street’s identification as a Main street (provided by MassGIS), and the nature of land usage (a seven-group typology based on a cluster analysis of the representation of each land use).

### Analysis

We ran all models using the felm command in the lfe package in R [49]. An initial set of models examined the association between street- and tract-level violence and test scores without considering individual-level fixed effects. These models took the form

$$Y_{it}=\beta_{1}X_{it}+\beta_{2}X_{jt}+\beta_{3}X_{kt}+\alpha_{school}+\alpha_{year}+\alpha_{grade}+\mu_{it}$$

where *Y*_*it*_ is the outcome measure (e.g., Math test score) for student *i* in year *t*; each β is a vector of estimated parameters for the effect of attributes of student *i* (*X*_*it*_; including demographics and programmatic assignment (i.e., English Language Learner status, Special Education eligibility), street *j* (*X*_*jt*_; including violence level and predominant land usage), and census tract *k* (*X*_*kt*_; see measures described above) in year *t*; and each α is a set of fixed effects for schools, school year, or grade level (to adjust for differences in the scaling of tests across grade levels and years). Because the students are nested in streets and the streets nested in tracts, a multilevel model would be an appropriate alternative (because some street segments form the boundary between tracts, we create strict nesting by attributing each segment to the census tract in which the majority of its land parcels sit). In order to mimic a multilevel model, we center the level of violence on a street relative to all streets in that tract in a given year; this is the only continuous variable in the model at the individual or street level and thus is the only variable for which this is necessary. Additionally, to partially account for the nesting effects that would be handled more formally by multilevel models, the models included clustered standard errors for streets and census tracts. Note that it was not possible to run multilevel models in the traditional form of time point nested in individual nested in geography. This specification requires that all time varying predictors pertain to the lowest level of analysis (in this case students); preictors at higher levels are assumed to be time invariant. The data here violate this assumption as the primary hypotheses regard shifts in the crime level of streets and neighborhoods.

The fixed effects models took the form

$$Y_{it}=\beta_{1}X_{it}+\beta_{2}X_{jt}+\beta_{3}X_{kt}+\alpha_{school}+\alpha_{year}+\alpha_{grade}+\alpha_{i}+\mu_{it}$$

where the addition of α_i_ are fixed effects for each student *i*. The only other difference from the previous models was the removal of time-invariant measures (i.e., the removal of certain elements in *X*_*jt*_, *X*_*jt*_, and *X*_*kt*_, including student sex, race; descriptors of street land usage which would generally be time invariant were included to account for students who lived on more than one street over the three-year period).

For all models we pooled student scores from all years into two samples, one for each subject test and limited to those who had taken that subject test in two or more years (amounting to 57,651 Math tests taken by 23,380 students, and 57,091 ELA tests taken by 23,155 students). It is worth noting that, while fixed effects models are one of the best ways to measure changes in outcomes owing to different experiences over time (or what one might call “treatments”), in this case exposure to violent crime, their power to detect effects are limited by the sample size and amount of underlying variation in said treatments. To this point, 47% of students included in the final analysis had three time points as opposed to two, increasing the potential for variation. Further, nearly all students in the sample (82%) experienced a change in the amount of violent crime on their street, suggesting sufficient variance to estimate an effect of changes in exposure to crime, even in a fixed effects model.

### Human subjects research

The authors affirm that the study was approved by the Northeastern University Institutional Review Board and use of data was permitted by the Boston Public Schools Office of Data and Accountability.

## Results

### Sample composition

Table 1 describes the characteristics of the students analyzed in the study, as well as of the streets and neighborhoods where they lived; here we focus on the full sample rather than the subsample that took tests in multiple years. Like many similar urban districts, BPS is a majority-minority district, which was reflected in those taking standardized tests. Nearly equivalent numbers of Black and Latinx students were in our sample (37% and 38%, respectively). About three-quarters (77%) of students were living in poverty (i.e., eligible for free or reduced-price meals under traditional criteria), and 42% were either currently or formerly in English-Language Learning (ELL) programs. These proportions were slightly different from the full school population, which was 44% Latinx, 33% Black, 72% living in poverty, and 42% currently or formerly involved in ELL programs in 2013. MCAS tests are graded from 200–280, with a score of 240 reflecting the cut-off between “Needs Improvement” and “Proficient.” The average score for both subjects was 238, but Math scores varied more markedly (*sd* = 19.19 vs. 15.56). About half of students qualified as Proficient in each test (ELA: 51%; Math: 46%).

### Student residential context and community violence

The average street with student residents had just under 3 violent events (*mean* = 2.94) in a single year, though this was due to a heavy skew from outliers. In any given year, only 39–41% of streets had any violent events. We considered two thresholds for defining hotspots. We first adopted the most modest definition of a hotspot: any street with 2 or more violent events in a year, amounting to 27–29% streets in any given year. Using the more stringent threshold of 6 violent events in a year (i.e., about 1 every 2 months or more), 10–11% of streets suffered chronically from crime.

Consistent with the district’s demographic composition, students were more likely to live on hotspot streets and in neighborhoods with above average crime than would be expected by chance. Across years, 58% of students lived in a neighborhood with above average crime. Meanwhile, 67% of students lived on a street that was a low-threshold hotspot (i.e., 2 or more violent events) the year that they took an MCAS test; the same proportion was 44% for high-threshold hotspots. Unsurprisingly, students living in poverty were even more likely to live in high-crime contexts. 63% of students living in poverty lived in a neighborhood with above-average crime, 74% lived on a low-threshold hotspot, and 50% lived on a high-threshold hotspot. The same proportions were 39%, 44%, and 24% for those not living in poverty.

**Fig 1:**
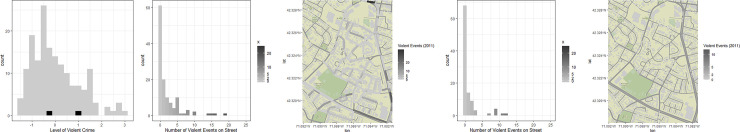
The variation in crime exposure within and between neighborhoods. (a) The distribution of the level of violent crime for all census tracts with student residents, highlighting two neighborhoods, one with below-average the other with above-average violent crime. Below is a direct comparison of the distribution of violent events across the streets of the two neighborhoods (b, d; above-average crime neighborhood on top) and corresponding maps (c, e). Note that scales for numbers of crimes (*x*-axis and coloration) were kept consistent between the two tracts to enable easier comparison.

Unsurprisingly, students living in neighborhoods with above-average crime rates were more likely to live on streets with violent crime. 76% of such students resided on a low-threshold hotspot and 51% on a high-threshold hotspot, meaning only 24% of students lived on streets with little to no crime. Conversely, per the insight from criminology of place that there are high-crime streets even in low-crime neighborhoods, 56% of students living in neighborhoods with below-average crime lived on low-threshold hotspots and 34% lived on high-threshold hotspots. Thus, there were students in high-crime neighborhoods living on low-crime streets and students in low-crime neighborhoods living on high-crime streets. This is also illustrated in Fig 1, which shows the distribution of violent crime events in 2011 across the streets of one neighborhood with above-average violent crime and another with below-average violent crime. As can be seen there, both neighborhoods have a mix of high-crime, low-crime, and no-crime streets, albeit with more of the latter in the below-average crime neighborhood.

### Baseline models: Street and neighborhood crime and academic achievement

Before proceeding to the full analysis, we confirmed that there was sufficient street- and tract-level variation in academic achievement to justify analyzing effects of events at each geographic scale (intraclass correlation coefficients of 1.5–3% and all *p*-values < .05 at both levels when accounting for individual-level demographic characteristics; calculated using multilevel models in R package lme4 [50] separately for each year to avoid repeated time points for many students). We ran models in two stages. First, we ran a set of regression models with no individual-level fixed effects. These were intended to identify the baseline association between violence in street and neighborhood contexts and academic achievement while controlling for contemporaneous correlates. We then incorporated individual-level fixed effects, leveraging repeated measures to account for unobserved characteristics that might be partially responsible for these baseline associations (see next subsection). Models included a variety of student-, street segment-, and census tract-level factors (see Methods for more detail on predictors; all parameter estimates reported in Table 2).

**Table 2:** Complete parameter estimates from linear models predicting scores on MCAS tests across grades and school years, based on characteristics of the student and street and tract of residence, with and without student-level fixed effects.

	Math				ELA			
	(1)	(2)	(3)	(4)	(1)	(2)	(3)	(4)
Student Characteristics								
*Male*	0.71***	0.71***	0.71***	—	-2.79***	-2.78***	-2.78***	—
	(0.17)	(0.17)	(0.17)	—	(0.12)	(0.12)	(0.12)	—
*Race*^a^								
Asian	13.87***	13.89***	13.86***	—	5.45***	5.46***	5.44***	—
	(0.44)	(0.44)	(0.44)	—	(0.38)	(0.38)	(0.38)	—
Latinx	1.51***	1.51***	1.51***	—	1.05***	1.05***	1.05***	—
	(0.25)	(0.25)	(0.25)	—	(0.21)	(0.21)	(0.21)	—
Mixed / Other	3.93***	3.93***	3.92***	—	3.43***	3.44***	3.43***	—
	(0.69)	(0.69)	(0.69)	—	(0.52)	(0.52)	(0.52)	—
Native Amer.	0.89	0.90	0.92	—	1.42	1.43	1.44	—
	(1.71)	(1.71)	(1.72)	—	(1.23)	(1.24)	(1.24)	—
White	6.65***	6.63***	6.61***	—	4.53***	4.51***	4.50***	—
	(0.44)	(0.44)	(0.44)	—	(0.36)	(0.36)	(0.36)	—
Free/Reduced Lunch	-3.88***	-3.87***	-3.86***		-3.43***	-3.42***	-3.41***	
	(0.28)	(0.27)	(0.27)		(0.25)	(0.25)	(0.25)	
*ELL Status*								
Current	-6.10***	-6.10***	-6.10***	-0.29	-8.48***	-8.48***	-8.48***	4.18*
	(0.27)	(0.27)	(0.27)	(2.26)	(0.23)	(0.23)	(0.23)	(2.28)
Former	5.71***	5.70***	5.70***	0.63	4.00***	4.00***	4.00***	5.88**
	(0.28)	(0.28)	(0.28)	(2.34)	(0.24)	(0.24)	(0.24)	(2.36)
*Special Ed*.	-11.18***	-11.18***	-11.18***	0.69*	-10.83***	-10.82***	-10.82***	-0.83**
	(0.25)	(0.25)	(0.25)	(0.39)	(0.19)	(0.19)	(0.19)	(0.35)
Street Characteristics								
*Count of Violent Events*	-0.27***	—	—	—	-0.19***	—	—	—
	(0.09)	—	—	—	(0.06)	—	—	—
*Low Hotspot (> = 2 Events)*	—	-0.92***	-0.64***	0.02	—	-0.66***	-0.51***	0.01
	—	(0.17)	(0.18)	(0.18)	—	(0.13)	(0.15)	(0.15)
*High Hotspot (> = 6 Events)*	—	—	-0.49**	—	—	—	-0.27*	—
	—	—	(0.20)	—	—	—	(0.16)	—
*Main*	0.17	0.20	0.19	-0.37	-0.14	-0.11	-0.12	0.07
	(0.22)	(0.22)	(0.22)	(0.28)	(0.16)	(0.16)	(0.16)	(0.23)
*Predominant Zoning*^b^								
3-Family Mixed	-0.63**	-0.47	-0.45	0.06	-0.66**	-0.54*	-0.53*	-0.23
	(0.32)	(0.32)	(0.32)	(0.40)	(0.29)	(0.29)	(0.29)	(0.43)
2-Family w/ single-family	-0.33	-0.26	-0.26	0.44	-0.36	-0.31	-0.31	-0.05
	(0.36)	(0.36)	(0.36)	(0.48)	(0.31)	(0.32)	(0.32)	(0.45)
Pure Commercial	-0.43	-0.44	-0.35	1.19	-0.42	-0.41	-0.37	0.72
	(0.49)	(0.50)	(0.49)	(0.89)	(0.42)	(0.43)	(0.42)	(0.82)
Exempt	-1.28***	-1.41***	-1.28***	0.31	-1.13***	-1.22***	-1.15***	-0.46
	(0.44)	(0.39)	(0.40)	(0.69)	(0.37)	(0.35)	(0.36)	(0.66)
Condominiums	-0.14	0.04	0.04	0.39	0.12	0.25	0.25	-1.06
	(0.51)	(0.51)	(0.50)	(0.79)	(0.42)	(0.42)	(0.42)	(0.75)
Mixed Commercial	-1.05**	-0.97**	-0.89**	0.72	-1.44***	-1.38***	-1.34***	-0.29
	(0.44)	(0.41)	(0.42)	(0.66)	(0.40)	(0.38)	(0.39)	(0.70)
Tract Characteristics								
*Violent Crime*	-0.57***	-0.54***	-0.54***	-0.30***	-0.45***	-0.43***	-0.43***	-0.09
	(0.15)	(0.15)	(0.15)	(0.11)	(0.11)	(0.11)	(0.11)	(0.15)
*Socioeconomic Status*	-0.35**	-0.33**	-0.30*	0.02	-0.35***	-0.34***	-0.32**	0.03
	(0.17)	(0.16)	(0.16)	(0.17)	(0.13)	(0.13)	(0.13)	(0.17)
*Adult Acad*. *Attainment*	0.35*	0.30	0.32	0.08	0.35**	0.32**	0.33**	-0.05
	(0.20)	(0.20)	(0.20)	(0.24)	(0.16)	(0.15)	(0.15)	(0.22)
*Physical Disorder*	0.06	0.14	0.15	0.08	-0.09	-0.03	-0.02	-0.08
	(0.26)	(0.26)	(0.26)	(0.26)	(0.18)	(0.18)	(0.18)	(0.22)
*Custodianship*	0.01	-0.02	-0.03	-0.09	-0.14	-0.16	-0.17	-0.15
	(0.18)	(0.17)	(0.17)	(0.17)	(0.15)	(0.15)	(0.15)	(0.13)
Grade-Level Fixed Effects	Yes	Yes	Yes	Yes	Yes	Yes	Yes	Yes
School Year Fixed Effects	Yes	Yes	Yes	Yes	Yes	Yes	Yes	Yes
Student Fixed Effects	No	No	No	Yes	No	No	No	Yes
**Records (Students/ Streets/Tracts)** ^**c**^	**57,651 (23,380/5,544/164)**				**57,043 (23,142/5,529/164)**			

*Note*: Sex and race were included in the initial models but were omitted with the inclusion of individual-level fixed effects because they were time invariant. Street-level variables that would appear to be time invariant (e.g., street classification) were included because some students lived on more than one street over the three school years.

^a^-A series of dichotomous variables reflecting a student’s race, with Black as the reference category.

^b^-A series of dichotomous variables reflecting a street’s predominant land usage, based on a cluster analysis of land use types. Single-Family Residential is the reference group.

^c^-Students are not perfectly nested in streets and tracts both because street- and tract-level measures vary with time and some students may have lived on more than one street over the three school years. In place of multilevel models, clustered standard errors were included at these two geographic levels.

*—*p* < .05

**—*p* < .01

***—*p* < .001.

The initial models found that higher crime at the neighborhood and street levels significantly predicted lower scores on both subject tests (see Model (1) for both Math and ELA in Table 2). Neighborhood had a modestly stronger effect on math scores (Math: B = -0.57, *p* < .001; ELA: B = -0.45, *p* < .001), though this difference was non-significant. Given that the neighborhood measure of crime is a standardized measure with a range of ~4.5, the final estimates translate to ~2.6 points (3.2% on the 80 point scale) and ~2 points (2.5%) of difference between the highest and lowest crime neighborhoods on Math and ELA tests, respectively.

The count of violent events on a street also predicted lower scores on both subject tests (Math: B = -0.27, *p* < .001; ELA: B = -0.19, *p* < .001). In order to better quantify the effect of living on a high-crime street, we ran a second set of models that found that students living on a “low” hotspot (2 violent events or more in a year) scored just under 1 point lower on tests (Math: B = -0.92, *p* < .001; ELA: B = -0.66, *p* < .001; see Model (2) for both Math and ELA in Table 2). A final set of models distinguished between low- and high-threshold hotspots (see Model (3) for both Math and ELA in Table 2). These found that living on a low-threshold hotspot street was associated with test scores ~.5 pts. lower than others living in the same neighborhood (Math: B = -0.64, *p* < .001; ELA: B = -0.51, *p* < .001), and living on a high hotspot street had an additional effect of ~.3-.5 pts. (Math: B = -0.49, *p* < .001; ELA: B = -0.27, *p* < .05), for a total of just under 1 pt. of difference (~1.25%).

Though these results are substantial, it is worth evaluating their sensitivity to confounders, including those that would accompany spatial segregation [using 51]. We find that street-level coefficients were particularly susceptible to such confounders. If one or more confounders accounted for 1.0% - 1.9% of the variance in the street-level crime and test scores (depending on the specific combination of predictor and outcome), the results would be rendered non-significant. For point of comparison, this would be equivalent to 1–2 times the relationship of eligibility for free-or-reduced lunch. The results at the neighborhood level were somewhat more robust (1.8% of shared variance with a confounder to render results non-significant, though this was equivalent to 5-times the effect of eligibility for free-or-reduced lunch, given a lesser correlation with neighborhood-level violence).

### Fixed-effects models: Controlling for individual characteristics

We ran the fixed effects model only measuring street-level violence as either a low threshold hotspot or not being that this was the simplest and most interpretable model from the initial analysis. All parameters are reported in Model (4) for both Math and ELA in Table 2. The introduction of the fixed effects eliminated the effects of street-level crime when measured as a low-threshold hotspot (Math: B = 0.02, *p* = *ns*; ELA: B = 0.01, *p* = *ns*). Per the note in the Analysis subsection regarding variability, 23% of students resided on a low hotspot and a non-hotspot at different points during the study period. This is considerably smaller than those who saw any change in crime on their street but results in >5,000 individuals for both Math and ELA, which is still sufficient variation to detect an effect. Accordingly, the diminishment of magnitude and significance was the same if we measured street-level crime as log-transformed counts of events (not reported in the Table; Math: B = 0.03, *p* = *ns*; ELA: B = -0.01, *p* = *ns*). The violent crime rate for the neighborhood continued to predict lower performance on Math tests (B = -0.30, *p* < .001), but not on ELA tests (B = -0.09, *p* = *ns*).

Last, we considered whether student mobility might be confounding results in some way. There is often considerable mobility in inner-city student samples [e.g., 52], and the same is true in our data set as 14% of students had two or more streets of residence over the study period. Though this is not an overwhelming amount of the sample, it could undermine interpretations. For robustness purposes we re-ran all analyses excluding these individuals. All results were the same (see S1 File).

## Discussion

The analysis demonstrated that community violence has independent relationships with academic achievement at the street and neighborhood levels. This story is true as our findings progress from exposure to baseline association to more rigorous testing through fixed effects models. First, exposure to community violence was correlated across geographic scales, but living in a high-crime neighborhood did not always equate to living on a hotspot, or vice versa. Indeed, as illustrated in Fig 1, students who lived in high-crime neighborhoods were more likely to live on hotspot streets, but a substantial proportion of students lived on low-crime streets in such neighborhoods (24%). Even more striking, 56% of students living in low-crime neighborhoods resided on streets with at least two violent events. These findings indicate that a precise understanding of exposure to community violence requires measurement at each of these scales.

Parts two and three of the analysis evaluated to what extent community violence at the street and neighborhood level might directly impact academic achievement. In both, neighborhood was more predictive of performance. There was ~3% difference between the highest and lowest crime neighborhoods, compared to ~1.5% difference between those living on high hotspot streets and their neighbors living on non-hotspots (based on a range of a range of ~4.5 standard deviations for the neighborhood violence measure, multiplied by the parameter estimate, and an 80-point grading scale). Put another way, we might note that the difference between living on a high hotspot street and a non-hotspot is equivalent to the difference between living in a census tract that is moderately above average in crime and living in another that is moderately below average (defined as 1 standard deviation from the mean). The two effects were additive, meaning that a student living in the neighborhood with the highest level of crime performed on average 3% worse on standardized tests than a classmate in a neighborhood with the lowest level of crime, but living on a hotspot street in that same neighborhood predicted an approximately 1.25% *further* drop in scores—a total of a ~4.5% deficit in relation to classmates whose residential context was free of violence. This is particularly important to consider when we recall that many students are exposed to community violence at the one scale and not the other.

The incorporation of student-level fixed effects, however, indicated that these associations were largely due to spatial segregation of families with lower performing students being more likely to reside on hot spot streets and in high crime neighborhoods. This was particularly salient for the effect of crime at the street level. Meanwhile, neighborhood-level violent crime was still predictive of lower Math test scores, albeit with attenuated differences of ~1.5% between the highest and lowest crime neighborhoods. This diminishment is to be expected given the more rigorous design and the limited amount of change in a student’s test scores from year to year. The robustness of this relationship was the exception, though, as neighborhood violent crime was no longer predictive of ELA scores nor was street-level crime predictive for *either* test subject. This stands in partial contrast to previous research, though the differences might not be as stark as they seem. First, though many studies have observed relationships between neighborhood-level community violence and both test subjects, only one to our knowledge has used student-level fixed effects [5]. Another study with a natural experiment design only identified an effect on ELA tests [4], in contrast to the unique effect on Math tests here. As such, our split result is curious but not without precedent and may point to small or variable effect sizes that are visible in some studies and not others [53]. Indeed, the maximum effect size of neighborhood violence on Math test performance in the final model was approximately 0.1 standard deviations, which is actually about the same size or larger than other findings looking at standardized test scores [4, 5]. Second, a handful of studies have found an effect of violent events on performance to a test or cognitive assessment occurring within 1–2 weeks thereafter [4, 12, 37]. The distinct research design used in those studies, however, assessed the acute effect of a community violence event on academic performance, whereas the current study evaluates the overall or cumulative impact of community violence over the year. An alternative interpretation might be that living long-term on high-violence streets could have persistent effects on academic achievement that are not especially sensitive to short-term increases in crime. That is to say, the effects of violence could have long-term impacts on growth, especially when students reside on hot spot streets or high crime neighborhoods for many years, but these would not be observable through a natural experiment designed that concentrates on short-impacts of exposure [see for example 5].

Our study did not directly test the mechanisms by which community violence could impact academic achievement, but the findings might be useful in guiding future work on this question. As noted at the outset, prevailing models for how community violence impacts academic achievement include trauma from direct or indirect exposure and socialized behavioral tendencies. If direct exposure or knowledge of violent crime events were primary factors in diminishing academic achievement throughout the course of the schoolyear, then we might have expected crime on one’s street to maintain its independent effect. It is not to say that crime on one’s street would need to be more impactful than the neighborhood to support the trauma interpretation, but that students would be most likely to directly experience or gain knowledge of events closer to their homes. The sole effect of neighborhood-level community violence across the year instead suggests the operation of social processes that operate at this broader scale [28, 29].

The results consistently pointed to a strong role for spatial segregation driving the community violence-academic achievement relationship—specifically, the existence of one or more unobserved variables that are correlated with both the tendency of a family to live in a high crime area and the lower academic performance of its children. This was especially true at the street level, which is a novel observation. It is well established that families are often constrained when selecting a neighborhood to live in, either by financial resources, discrimination, the desire to live near friends and family, or other systemic or personal limitations [32–35, 54]. These tendencies can then be associated with unobserved characteristics that lead to other outcomes, including academic achievement. But the same has not been demonstrated for individual streets within neighborhoods. In this study we see that students who live on high-crime streets within neighborhoods were more likely to score lower than their neighbors on standardized tests, but that this was not because of the crime itself. Instead, some unobserved set of features belonging to these families is leading to both the tendency to live on more violent streets and to perform lower in school. There are numerous potential hypotheses for why this might be the case, including the location of subsidized housing in high-crime neighborhoods, the lack of knowledge or resources to find housing on safer streets, or other personal characteristics. Testing these explanations are beyond the scope of the current study and data. That said, it is still feasible that living on a high-crime street could have long-term impacts on academic achievement that are not visible in fixed effects models, which only assess the impacts of year-to-year changes in crime.

### Limitations and conclusion

The study has multiple limitations that call for future research. First, though Boston is representative of many urban centers in America, with its majority-minority student population that varies widely in its exposure to violence, it will be important to replicate this work in other cities. Second, given the inconsistent findings regarding the community violence-academic achievement relationship in this and other studies (e.g., one test subject or geographic scale and not another), it is critical that additional replications be conducted, enabling a more robust evaluation of its strength. In doing so, these replications should consider other forms of measurement for both community violence and academic achievement. For the former, 911 dispatches are largely constituent-reported, making them robust to processes of crime reporting, but also sensitive to local biases in what to report and not report [45, 55]. For the latter, statewide standardized tests are only one way to assess academic achievement, and one that can be racially and socioeconomically biased [56]. Other options include grades and cognitive tests. Further, future research might consider how long students have resided in high or low crime neighborhoods and hotspot streets to account for intensity or duration of exposure. Third, there is the open question of how families choose where they live within a neighborhood—including the structural limitations placed on this choice—and why these patterns would be correlated with the academic achievement of children. Rather than speculate here on those processes, we think this offers an exciting opportunity for future research.

Conceptually, two main findings from this study are seemingly disconnected. On the one hand, neighborhood- and street-level community violence are partially independent quantities that are not always experienced together. On the other hand, we found that only neighborhood-level community violence had robust consequences for academic achievement. This provides some useful guidance to policymakers while considering the impacts of exposure to community violence on its students. The most apparent lesson is that attention should focus on neighborhood-level patterns. That said, the observation that the geography of exposure to community violence exists at multiple scales, and that students in the same neighborhood can have distinct levels of exposure, could be meaningful for other studies—from trauma to socialization to even acute impacts on academic attainment, which we do not test here. It adds nuance to how we describe community violence in a given case [see 57 for a review]. We encourage others to pursue these questions moving forward.

## Supporting information

S1 File The attached file contains (1) expanded description of the methods used to develop multi-item, neighborhood-level measures, including weighting of items in scales, and (2) the results of a series of additional tests evaluating the robustness of the results. (DOCX)
