# Supplementary material for: Community violence and academic achievement: High-crime neighborhoods, hotspot streets, and the geographic scale of “community”
Source: PLoS One. 2021 Nov 10;16(11):e0258577. doi: 10.1371/journal.pone.0258577 (PMC8580231; doi:10.1371/journal.pone.0258577)
Supplement: S1 File — (DOCX) [file pone.0258577.s001.docx]

**Supplementary Online Materials**

**Methods**

*Measures*

To measure violent crime on streets and in neighborhoods, we combined three indices of violence: *private conflict* arising from personal relationships (e.g., domestic violence); *public violence* that did not involve a gun (e.g., fight); and *prevalence of guns*, as indicated by shootings or other incidents involving guns. Each of these were based on data groupings of case types developed through factor analyses in previous work ([O'Brien and Sampson 2015](#_ENREF_1); [O'Brien, Sampson and Winship 2015](#_ENREF_3)). The case types and their frequencies in an example year are reported in Table S1.

We also drew on BARI’s Boston Data Portal for a series of tract-level covariates known to be related to academic achievement. In nearly all cases these are multi-measure constructs intended to measure an overarching aspect of a neighborhood. BARI and BPS constructed them as an effort to specify the sources of geographic inequities in academic achievement ([O’Brien, Hill and Contreras 2017](#_ENREF_4)). These include socioeconomic status (e.g., median household income), academic attainment of adults (e.g., proportion of adults with Bachelor’s degree), physical disorder (i.e., “broken windows”; O'Brien, Sampson and Winship 2015), and custodianship ([i.e., the tendency of residents to attend to issues in public spaces; O'Brien, Sampson and Winship 2015](#_ENREF_3)). Table S2 lists the measures included in each of these constructs and their weighting. Work from this earlier project also examined the role of public health (e.g., premature mortality), proportion immigrant, and residential stability (e.g., median years living in the neighborhood), but found them to have no predictive power independent of the other factors. For this reason we omit them in the analysis presented here.

**Results from Robustness Tests**

We also ran two robustness tests, one regarding street length and another regarding residential mobility. The results are reported in Table S3. For street segments, one might argue that individuals are not directly exposed to all violence on their street if they live on an exceptionally long street segment wherein a student living on one end of the street might perceive limited direct threat from a violent event on the other end of the street. This should not be a major concern for our data set, as Boston’s street segments are rather short (median in this study = 94 m). To confirm, we set a cutoff point of 250 m (i.e., the average student would live a maximum of 125 m from any event on the street), which might be far enough to be considered outside the immediate surroundings of their home. This includes 6% of streets in the study and 14% of students. We re-ran the models excluding individuals whose streets were above the cutoff and found the same all results to be the same.

For residential mobility, there is often considerable mobility in inner-city student samples ([e.g., O'Brien, Gallup and Wilson 2011](#_ENREF_2)), and the same is true in our data set as 14% of students had two or more streets of residence over the study period. Though this is not an overwhelming amount of the sample, it could undermine interpretations. For robustness purposes we re-ran all analyses excluding these individuals. All results were the same.

**Table S1. Frequency of 911 Call Types at Addresses by Category in 2011**

| **Case Type** | **Count (2011)** |  | **Case Type** | **Count (2011)** |
| --- | --- | --- | --- | --- |
| **Private Conflict (911)** | |  | **Public Violence (911)** | |
| Breaking and entering in progress | 1426 |  | Assault and battery in progress | 2181 |
| Landlord/tenant trouble | 667 |  | Assault and battery report | 1565 |
| Vandalism report | 3502 |  | Armed robbery | 350 |
| Violation of restraining order | 972 |  | Emotionally disturbed person: violent or injured | 5896 |
|  |  |  | Fight | 4623 |
| **Prevalence of guns (911)** |  |  | Person with knife | 687 |
| Assault and battery with deadly weapon | 85 |  |  |  |
| Person with a gun | 625 |  |  |  |
| Shots fired | 1009 |  |  |  |
| Person shot | 50 |  |  |  |

**Table S2.** Items used to measure neighborhood constructs, their sources, definitions, and weighting in the final metrics.

| **Indicator** | **Source** | **Definition** | **Weight** |
| --- | --- | --- | --- |
| *Adult Academic Attainment* | |  |  |
| Postsecondary attainment | ACS | Percent of census tract adults (25 years and older) who have earned a Bachelor’s degree or higher | 1.0 |
| *Custodianship* |  |  |  |
| Custodianship | 311 reports ([O'Brien, Sampson and Winship 2015](#_ENREF_3)) | The likelihood that residents will use 311 to call in an issue in the public domain (e.g., pothole) | 1.0 |
| *Violent Crime* |  |  |  |
| Gun use | 911 reports ([O'Brien and Sampson 2015](#_ENREF_1)) | Rate of events that involve the use of guns (e.g., shooting). | .89 |
| Private conflict | 911 reports ([O'Brien and Sampson 2015](#_ENREF_1)) | Rate of events that reflect interpersonal conflict in the neighborhood (e.g., domestic violence). | .88 |
| Public violence | 911 reports ([O'Brien and Sampson 2015](#_ENREF_1)) | Rate of events that reflect interpersonal violence that do not involve a gun (e.g., fight). | .88 |
| *Physical Disorder* | |  |  |
| Public Denigration | 311 reports ([O'Brien, Sampson and Winship 2015](#_ENREF_3)) | Disrespect for public space (e.g., graffiti, inappropriate disposal of trash). | 1.0 |
| Private Neglect | 311 reports ([O'Brien, Sampson and Winship 2015](#_ENREF_3)) | Deterioration to or misuse of privately-owned buildings and spaces (e.g., illegal rooming house). | 1.0 |
| *Socioeconomic Status* | |  |  |
| Family poverty | ACS | Rate of poverty at the Census Tract level | .88 |
| Median household income | ACS | Median household income of Census Tract | -.85 |
| Public assistance | ACS | Rate of receipt of SNAP benefits at the Census Tract level | .85 |
| Unemployment | ACS | Rate of unemployed individuals 16 years of age and older at the Census Tract level | .84 |

*Note:* ACS – American Community Survey; 311 – Requests for non-emergency government services; 911 – Requests for emergency government services. Measures drawn from ACS indicators use the five-year estimate whose median year is the desired year (e.g., 2009-2013 for 2011). Other measures are based on records from that calendar year.

**Table S3.**

Parameter estimates from linear models for the effects of street- and tract-level violence on MCAS tests across grades and school years from robustness tests.

|  | **Street Length <250m** | | | | | **No Residential Moves** | | | | | | | |  |
| --- | --- | --- | --- | --- | --- | --- | --- | --- | --- | --- | --- | --- | --- | --- |
|  | Math | | ELA | | | | Math | | | ELA | | |  |  |
|  | w/o FE | w/FE | w/o FE | | w/FE | | w/o FE | | w/FE | w/o FE | | w/FE |  |  |
| *Count of Violent Events (Street)* | -0.26^**^ | 0.02 | -0.18^**^ | | 0.02 | | -0.24^***^ | | 0.03 | -0.16^*^ | | -0.02 |  |  |
|  | (0.09) | (0.11) | (0.07) | | (0.08) | | (0.09) | | (0.11) | (0.07) | | (0.08) |  |  |
|  |  |  |  | |  | |  | |  |  | |  |  |  |
| *Low Hotspot Street (>=2 Events)* | -0.92^***^ | 0.04 | -0.64^***^ | | -0.07 | | -0.86^***^ | | 0.06 | -0.59^***^ | | 0.02 |  |  |
|  | (0.18) | (0.19) | (0.13) | | (0.16) | | (0.18) | | (0.18) | (0.13) | | (0.13) |  |  |
|  |  |  |  | |  | |  | |  |  | |  |  |  |
| *Violent Crime (Nbhd)*^a^ | -0.59^***^ | -0.39^***^ | -0.42^***^ | | -0.12 | | -0.41^***^ | | -0.29^***^ | -0.39^**^ | | -0.12 |  |  |
|  | (0.16) | (0.12) | (0.12) | | (0.15) | | (0.16) | | (0.12) | (0.12) | | (0.14) |  |  |
|  |  |  |  | |  | |  | |  |  | |  |  |  |
| **Records**  **(Omitted)** | **50,617**  **(7,034)** | | | **50,150**  **(6,893)** | | | | **49,020**  **(8,631)** | | | **48,211**  **(8,832)** | | | |

^a^ – Effects for neighborhood violent crime drawn from model with count of violent events at the street level. Differences between this and the model with low hotspot as the measure of violence at the street level were negligible.

**References**

O'Brien, Daniel, and Robert J. Sampson. 2015. "Public and Private Spheres of Neighborhood Disorder: Assessing Pathways to Violence Using Large-Scale Digital Records." *Journal of Research in Crime and Delinquency* 52:486-510.

O'Brien, Daniel Tumminelli, Andrew C. Gallup, and David Sloan Wilson. 2011. "Residential mobility and prosocial development within a single city." *American Journal of Community Psychology* 50(1-2):26-36.

O'Brien, Daniel Tumminelli, Robert J. Sampson, and Christopher Winship. 2015. "Ecometrics in the age of big data: Measuring and assessing "broken windows" using administrative records." *Sociological Methodology* 45:101-47.

O’Brien, Daniel T., Nancy E. Hill, and Mariah Contreras. 2017. "The Opportunity Index: A data-driven tool for countering inequities in Boston Public Schools." Boston, MA: Boston Area Research Initiative.
